# Application of AutoFom III equipment for prediction of primal and commercial cut weight of Korean pig carcasses

**DOI:** 10.5713/ajas.18.0240

**Published:** 2018-07-26

**Authors:** Jung Seok Choi, Ki Mun Kwon, Young Kyu Lee, Jang Uk Joeng, Kyung Ok Lee, Sang Keun Jin, Yang Il Choi, Jae Joon Lee

**Affiliations:** 1 Swine Science and Technology Center, Gyeongnam National University of Science and Technology, Jinju 52725, Korea; 2 Korea Institute for Animal Products Quality Evaluation, Sejong 30100, Korea; 3 Dodram Pig Farmers Cooperative, Icheon 17405, Korea; 4 Dodram Pig Farmers Service Co., Ltd., Icheon 17405, Korea; 5 Dodram LPC Co., Ltd., Anseong 17533, Korea; 6 Department of Animal Resources Technology, Gyeongnam National University of Science and Technology, Jinju 52725, Korea; 7 Department of Animal Science, Chungbuk National University, Cheongju 28644, Korea; 8 Department of Food and Nutrition, Chosun University, Gwangju 61452, Korea

**Keywords:** AutoFom III, Ultrasound, Prediction, Primal Cuts, Calibration, Validation

## Abstract

**Objective:**

This study was conducted to enable on-line prediction of primal and commercial cut weights in Korean slaughter pigs by AutoFom III, which non-invasively scans pig carcasses early after slaughter using ultrasonic sensors.

**Methods:**

A total of 162 Landrace, Yorkshire, and Duroc (LYD) pigs and 154 LYD pigs representing the yearly Korean slaughter distribution were included in the calibration and validation dataset, respectively. Partial least squares (PLS) models were developed for prediction of the weight of deboned shoulder blade, shoulder picnic, belly, loin, and ham. In addition, AutoFom III’s ability to predict the weight of the commercial cuts of spare rib, jowl, false lean, back rib, diaphragm, and tenderloin was investigated. Each cut was manually prepared by local butchers and then recorded.

**Results:**

The cross-validated prediction accuracy (R^2^cv) of the calibration models for deboned shoulder blade, shoulder picnic, loin, belly, and ham ranged from 0.77 to 0.86. The R^2^cv for tenderloin, spare rib, diaphragm, false lean, jowl, and back rib ranged from 0.34 to 0.62. Because the R^2^cv of the latter commercial cuts were less than 0.65, AutoFom III was less accurate for the prediction of those cuts. The root mean squares error of cross validation calibration (RMSECV) model was comparable to the root mean squares error of prediction (RMSEP), although the RMSECV was numerically higher than RMSEP for the deboned shoulder blade and belly.

**Conclusion:**

AutoFom III predicts the weight of deboned shoulder blade, shoulder picnic, loin, belly, and ham with high accuracy, and is a suitable process analytical tool for sorting pork primals in Korea. However, AutoFom III’s prediction of smaller commercial Korean cuts is less accurate, which may be attributed to the lack of anatomical reference points and the lack of a good correlation between the scanned area of the carcass and those traits.

## INTRODUCTION

As the national income has been increasing in Korea, the annual consumption of meat per capita was 46.8 kg in 2015 including the consumption of pork (22.5 kg), beef (10.9 kg) and poultry (13.4 kg) [1]. The Animal Products Grading Service (APGS) in Korea has been promoted since 1989 and has led to many changes in production, distribution, and consumption of livestock products. The number of slaughtered pigs in 2016 was 16,545,492 heads, and the number of grading pigs was 16,524,269 heads (99.87%, grading rate), which was a noticeable increase compared to 1,807,087 heads in 1993 [2]. Implementing APGS contributes greatly to the development of the livestock industry, including the change from live animal to meat products, conversion from warm carcass to cold carcass distribution, prevention of meat quality degradation, reliability of distribution, reasonable price formation, etc. Consumers can choose meat of various kinds and quality corresponding to payment price, and select the appropriate cuts and grades suitable for the cooking purpose. In addition, the incidence of high-grade meats has steadily increased, and in terms of pigs, the production rate of high-quality pork increased owing to the increased accumulation of grading information. The data from the APGS also are very helpful for development of breeding and rearing technology.

Korean consumers have traditionally preferred to grill meat; hence, cuts with abundant fat such as pork belly and shoulder blade are popular among consumers, and low-fat cuts such as ham and loin are less popular. Thus, the price difference of pork is very large in Korea [3]. Recently, consumers are increasingly interested in health, so they prefer meat cuts with low fat content [4,5]. However, because the grade evaluation of pork is indicated only in the carcass quality (by carcass weight and backfat thickness) and meat quality (by subjective marbling level, meat and fat colors, degrees of maturity, and texture) in whole pig carcass after slaughtering, the weight of each pork primal cut is unknown until it has been separated into primals.

Therefore, if the proportion of the primal cuts in the pig carcass can be determined before separation of primal cuts from the whole carcass, not only would it be possible to economically classify the pig carcass suitable for use, uniform meat quality, and yield prediction, it would also be very useful in industrial applications such as animal breeding, feed nutrition, system of rearing, and distribution fields.

Worldwide, several techniques exist for analyzing pig carcasses using non-destructive methods including dual-energy x-ray absorptiometry (DXA), magnetic resonance imaging (MRI), computed tomography (CT), and ultrasound imaging (US). CT, MRI, and DXA showed higher accuracy than US, whereas the US has the advantage of being applicable to any carcass size, is reasonably priced, has no radiation, and is a real-time measurement [6].

On-line grading of pig carcasses by ultrasound technology is widely used throughout Europe. The advantageous are it is a non-invasive, fully automated technology, which enables rapid feedback to slaughter houses on lean meat percentage (LMP), primal yields, and other specific traits of individual carcasses. This information enables the slaughter houses to increase slaughter production efficiency, as sorting of carcasses becomes possible before the carcasses enter the cooling room. Frontmatec Smoerum A/S (formerly known as Carometec A/S, Smorum, Denmark) launched an ultrasound-based classification system called AutoFom III in 2009. Currently, it is used for pig carcass classification in more than 14 countries. At present, in Korea, there is no utilization of AutoFom III in the determination of pork grades. Therefore, it is necessary to establish criteria for verifying utility and to verify the reliability through these studies. In 2015, an AutoFom III was installed in the Dodram LPC slaughterhouse in Korea.

The purpose of the present study was to evaluate the ability of AutoFom III to predict various primal and commercial cuts in Korean slaughter pigs. The obtained prediction accuracy and prediction errors of the calibration models were validated against an independent dataset.

## MATERIALS AND METHODS

### Animals and primal cuts

The pigs used in the calibration test were LYD (Landrace× Yorkshire, F1×Duroc) crossbreds. The pigs were transported to the Dodram LPC in Anseong, Gyeonggi Province, Korea from June to November 2015. The pigs were slaughtered according to the Livestock Hygiene Control Act. Selection of carcasses was based on backfat thickness (<10 mm, 10 to 40 mm, >40 mm) and hot carcass weight (<75 kg, 75 to 105 kg, >105 kg). A total of 162 LYD pigs were selected for the calibration test. Table 1 presents the final distribution of selected pigs. Females and castrates were included in the 50:50 ratio for each sorting group. To validate the calibration models, 154 LYD pigs from 27 farms contracted with Dodram were randomly selected from May to July 2016. A total of 11 primals and commercial cuts (belly, shoulder blade, spare rib, back rib, loin, tenderloin, shoulder picnic, ham, jowl, false lean, and diaphragm) were manually dissected and the weight recorded. On the day of slaughter, all carcasses were scanned by the AutoFom III, the hot carcass weight was registered, and the selected carcasses were allocated to a specific beam in the cold room for overnight storage. The following day, the cuts were prepared according to the Livestock Hygiene Control Act (Standard of separation for pork parts) and a pre-defined cut test protocol was used as a reference. To minimize errors in the separation process of the primal cuts, two pre-trained workers participated in the cutting process. The preparation of cuts was carried out as follows. The carcass was divided into six primal parts (fore leg, rib, loin, belly, hind leg, and the tenderloin which was removed and weighed separately). The fore leg was separated into the following four commercial cuts: shoulder picnic, spare rib, shoulder blade, and jowl, then deboned and the weight of the individual cuts recorded. The loin part was separated into two commercial cuts (loin and false lean), the loin was deboned, and the weights recorded. The belly was separated into three commercial cuts (belly bone-out, back rib, and diaphragm). The hind leg was deboned, and the weight of the hind leg bone-out including shank was recorded.

### AutoFom III scanning

**Figure 1:**
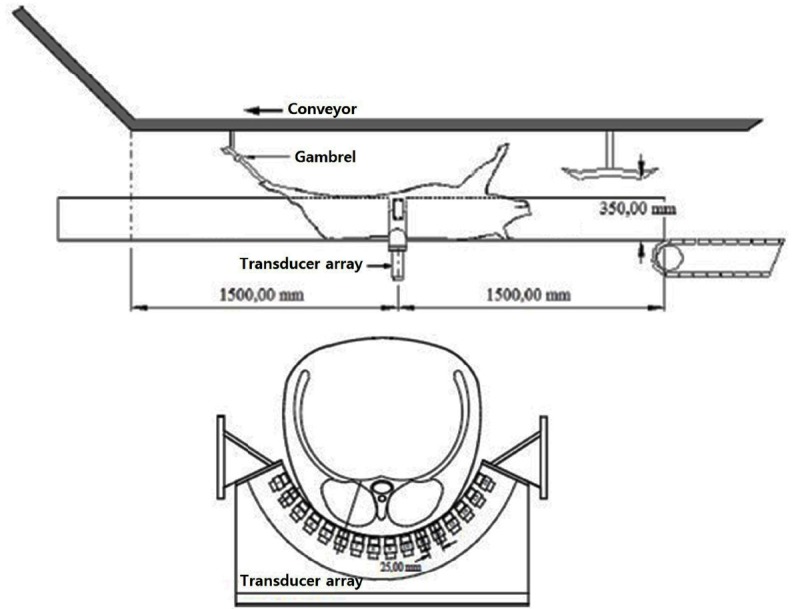
Carcass is pulled across an AutoFom III array with 16 ultrasound transducers. After scalding of pig carcass, pig carcass connected to the gambrel (RFID tag) of the conveyor passes through 16 ultrasound arrays mounted on the Autofom III chute, and the information of the carcass is input to the control computer.

The AutoFom III (Frontmatec Smoerum A/S, Denmark) equipment is installed just after the dehairing machine and the gambrel table prior to opening of the carcass. AutoFom III consists of a transducer array with 16 transducers (Figure 1), and the pig carcass is scanned on the back as the hind legs are pulled on the conveyor and passes over the ultrasound transducer array. The transducers are excited in turn with a repeated frequency of approximately 5 kHz (200 μs period) (Figure 1). Scanning of the carcass results in an ultrasound image and generation of 48 image parameters providing information on skin, fat, and lean measures. Hot carcass weight was included as parameter 49. The weight of the 11 primal cuts was used as reference for development of the prediction models. The prediction models were validated against the 2016 dataset. The root mean squares error of prediction (RMSEP) was calculated based on the AutoFom III predicted values and the reference values.

### Statistical analysis

The number of principal components (#PC) to be included in the models was established using principal component analysis (PCA). Partial least squares (PLS) regression was used to predict the weight of different cuts from the image parameters obtained during AutoFom scanning. The reference and AutoFom data were pre-processed using autoscaling, and the developed calibration models cross-validated using the leave-one-out approach. Variable selection was performed using interval partial least squares (iPLS) to optimize the PLS models. The PCA, PLS, and iPLS models were performed using the PLS Toolbox 8.2 (Eigenvector Research, Manson, IA, USA) with MatLab R2016b (MathWorks, Natick, MA, USA).

Prediction errors were evaluated by determining the root mean squares errors of calibration (RMSEC), root mean squares error of cross validation (RMSECV), and RMSEP. In addition, the prediction accuracy of the cross-validated calibration models (R^2^cv) and the prediction accuracy of validation data (R^2^pred ) set were determined. Furthermore, the bias between the average reference value and average AutoFom III predicted value was determined.

## RESULTS

Table 2 presents descriptive statistics for the 11 primal and commercial cuts. Among the 11 cuts, the heaviest cuts were deboned ham (8.70 kg) and deboned belly (6.31 kg). The weight of the remaining cuts is listed here in descending order: deboned shoulder picnic (4.14 kg), deboned loin (2.94 kg), deboned shoulder blade (2.29 kg), spare rib (1.86 kg), tenderloin (0.49 kg), back rib (0.44 kg), jowl (0.26 kg), false lean (0.20 kg), and diaphragm (0.13 kg).

Table 3 presents the AutoFom III calibration model statistics. Among the 11 individual cuts, the shoulder blade, shoulder picnic, loin, belly, and ham cuts had prediction accuracies greater than 0.77 R^2^cv values, and the highest R^2^cv value (0.86) was observed for the deboned belly cut. However, the tenderloin, spare rib, jowl, false lean, back rib, and diaphragm cuts had R^2^cv lower than 0.63, and the back rib cut had the lowest R^2^cv (0.34). The RMSECV values express the error to be expected when the AutoFom III predicted weight is compared to the manually prepared weight. The error will always be a sum of errors occurring when collecting the reference material and owing to equipment inaccuracy. Deboned ham had an RMSECV of 401 g. Deboned belly had an RMSECV of 352 g. The RMSECV was found in the diaphragm (14 g) and false lean (29 g) cuts. The RMSECV values should always be observed in relation to the average weight of the cut (Tables 2, 3) to reveal the level of magnitude of the error.

Table 4 presents the descriptive statistics including number of samples, average weight, and minimum and maximum weights of individual cuts for both the calibration (2015) and validation (2016) data set. The five cuts with prediction accuracy exceeding 0.77 are presented in Table 4.

**Figure 2:**
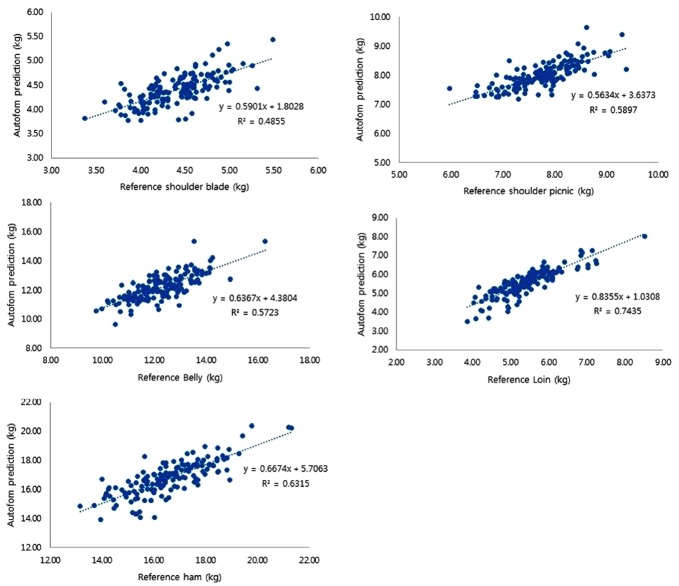
Graphs of linear regression analysis of AutoFom III predicted weights against manual dissection of five pork cuts (Shoulder blade, shoulder picnic, belly, loin, and ham) in the validation trial. The vertical axis is the predicted value by the Autofom III, and the horizontal axis is the actual dissected value. The regression equations of each meat were derived.

Table 5 and Figure 2 shows that the R^2^pred values of the validation test is lower than the R^2^cv in the five cuts. The highest R^2^pred value was 0.744 (deboned loin) and the lowest value (R^2^pred = 0.486) was found in the deboned shoulder blade. Comparing the prediction errors (RMSECV and RMSEP) reveals that for most of the validated cuts the errors are comparable. Numerically higher RMSEP values compared to the RMSECV values were observed in the shoulder picnic, loin, and ham cuts; however, these differences were considered minor.

## DISCUSSION

Since the early 2000s, several studies have been conducted to determine the cutting yield of pig carcasses by non-invasive methods [7–11]. Non-invasive prediction instruments are used to grade pig carcasses in European countries and North America. Brøndum et al [12] reported that the residual standard deviations (RSD) of AutoFom I were significantly lower than those of Fat-O-Meat’er (FOM) for ham (p<0.05), loin (p<0.03), and shoulder (p<0.01), and reported that the prediction error range of AutoFom I was 0.15 to 0.31 kg, whereas for FOM it was higher (0.18 to 0.46 kg). Busk et al [13] conducted a trial in Denmark and reported that the AutoFom I prediction of total LMP yielded a residual root mean squares error (RMSE) of 1.84 LMP. This was highly acceptable as the RMSE requirement to meet the European Union (EU) standards must be less than 2.5 LMP. The authors therefore concluded that the AutoFom I was very suitable for automatic prediction of LMP in pig carcasses. Fortin et al [14] reported that the prediction accuracy (R^2^) of AutoFom I predicted lean weights of the picnic, butt, loin, ham, and belly of Canadian pigs were 0.68, 0.59, 0.84, 0.80, and 0.60, respectively, and the RMSE values were 0.20 kg, 0.23 kg, 0.32 kg, 0.40 kg, and 0.35 kg, respectively. These results were similar to those of other grading equipment: HGP2 (Hennessy grading systems limited, Auckland, New Zealand), CVT-2 (Animal Ultrasound Services, Inc, Newyork, USA), and UltraFom 300 (SFK Technology A/S, Herlev, Denmark). Font i Furnols and Gispert [9] compared the accuracy of the FOM based on light reflectance, VCS2000 based on electromagnetic waves, and AutoFom I based on ultrasound for prediction of LMP, the AutoFom I and FOM showed lower RMSEP values (1.8% and 1.9%, respectively) than VSC2000 (e + V Technology GmbH, Oranienburg, Germany). The AutoFom I showed higher prediction accuracy (R^2^ = 0.78) than those of FOM and VCS2000 (0.77 and 0.70, respectively). Strzelecki et al [15] and Gispert et al [16] reported a 2.0% prediction error for AutoFom I, the prediction of the salable meat yield of AutoFom I showed an RMSE of 1.68% [14], and Brøndum et al [12] reported that the RSD (%) of AutoFom I predictions of total LMP of pig carcasses was 1.58% in Germany, 1.70% in the United States, and 1.84% in Denmark.

Lisiak et al [17] reported, that the regression coefficients for prediction of weight of untrimmed primal cuts (ham, loin, shoulder, belly) were 0.92, 0.87, 0.87, and 0.74, respectively using the Capteur Gras/Maigre (CGM, SYDEL, Lorient, France) equipment in polish commercial pigs.

Until now the previous studies presented above are related to AutoFom I performance. AutoFom III is far more advanced, and the image analysis software version has been modified and improved in AutoFom III compared to AutoFom I. This has decreased the prediction errors compared to AutoFom I; however, there are no scientific papers available on the performance of AutoFom III. To the best of our knowledge, the present study is the first to document the prediction results of 11 primal and commercial cuts of Korean pigs using AutoFom III.

No studies related to the prediction of the eleven cuts of this study was found, but, in the previous studies, the larger cuts showed a tendency for higher reliability than lighter cuts [7,18]. The reason for this is that the error of the weight may be greater in the cuts where the butcher has difficulties establishing anatomical fix-points, and hence it is difficult to prepare the cuts consistently [19].

In predicting the 11 cuts of this study using AutoFom III, the spare rib, back rib, jowl, false lean, and diaphragm cuts showed low prediction accuracies (R^2^cv<0.34). This means that only 34% of the variation can be explained by AutoFom III. Therefore, this indicates that AutoFom III cannot be used for the prediction of those 5 pig cuts.

For the verification of reliability of AutoFom III, the calibration model and validation model were compared. For loin and ham, some of the carcasses have a large deviation between the AutoFom predicted value and the reference value. Three observations (loin) and five observations (ham) were excluded from the respective datasets, as they were considered outliers for unknown reasons. This exclusion resulted in a decrease in RMSEP and an increase in R^2^pred (reported in Table 5) closer to the RMSECV and R^2^cv of the calibration dataset. It is concluded that the validation results are acceptable for both these cuts. The lower R^2^pred compared to R^2^cv values may be explained by a lower span and distribution of samples in the validation dataset compared to the calibration dataset. Small biases are observed; however, it is concluded that the biases are small compared to the mean weight of the cut (Table 5). It was expected that no biases appeared when the cut test protocol is similar for the calibration and validation test, and the same genetic lines are used.

In this study, the predictability of the AutoFom III in Korean slaughter pigs were verified. In general, high prediction accuracy was found in larger primal cuts (deboned shoulder blade, shoulder picnic, loin, belly, and ham). For the commercial cuts (spare rib, back rib, jowl, false lean, and diaphragm) the prediction accuracy was lower. In conclusion, AutoFom III is highly suitable for prediction of primal cuts in Korean slaughter pigs and hence acts as an advantageous management tool enabling the slaughterhouses to optimise their product sorting prior to the carcasses entering the cold room. Prediction of the weight of the smaller commercial cuts; jowl, false lean, back rib, and diaphragm is less accurate but may still be advantageous to the slaughterhouse depending on the bin sorting requirements.

## Figures and Tables

**Table 1:** Number and distribution of pigs selected according to backfat thickness and hot carcass weight for the AutoFom III calibration trial

Backfat thickness (mm)	Hot carcass weight (kg)					

	<75	75 – 85	86 – 95	96 – 105	>105	Sum
<10	-	-	-	-	-	
11 to 20	4	5	8			17
21 to 30	8	31	20	14	6	79
31 to 40	-	12	23	17	10	62
>40	-	-	-	1	3	4
Sum	12	48	51	32	19	162

**Table 2:** Descriptive statistics including number of observations, means, standard deviations (SD), and maximum and minimum values of 11 individual cuts of pigs used as reference data for the AutoFom III calibration trial

Cuts	N	Mean (kg)	SD (kg)	Min (kg)	Max (kg)
Shoulder blade bone-out	159	2.29	0.297	1.63	3.13
Shoulder picnic bone-out	157	4.14	0.471	3.14	5.27
Loin bone-out	157	2.94	0.426	2.01	3.92
Belly bone-out	159	6.31	0.93	4.05	8.95
Ham bone-out	161	8.70	1.01	6.30	11.34
Tenderloin	160	0.494	0.070	0.344	0.702
Spare rib	157	1.86	0.251	1.29	2.57
Jowl	157	0.255	0.037	0.170	0.358
False lean	156	0.203	0.041	0.126	0.332
Back rib	155	0.437	0.067	0.302	0.608
Diaphragm	155	0.130	0.019	0.088	0.178

**Table 3:** Calibration model statistics including cross-validated prediction accuracy and prediction errors (RMSEC and RMSECV) for AutoFom III calibration models of 11 commercial pig cuts

Cuts	# PC	N	R^2^cv	RMSEC (kg)	RMSECV (kg)
Shoulder blade bone-out	3	159	0.774	0.137	0.141
Shoulder picnic bone-out	2	157	0.818	0.197	0.200
Loin bone-out	3	157	0.849	0.159	0.165
Belly bone-out	3	159	0.856	0.340	0.352
Ham bone-out	3	161	0.840	0.389	0.401
Tenderloin	3	160	0.624	0.042	0.043
Spare rib	3	157	0.574	0.159	0.164
Jowl	3	157	0.456	0.027	0.027
False lean	3	156	0.497	0.028	0.029
Back rib	1	155	0.339	0.054	0.055
Diaphragm	2	155	0.503	0.013	0.014

#PC, number of principle components in the models; R^2^cv, cross-validated prediction accuracy; RMSEC, root mean squares error of calibration; RMSECV, root mean squares error of cross validation.

**Table 4:** Descriptive statistics including number of samples, average weight, and minimum and maximum weights of individual cuts for both the calibration and validation data set

Cuts (bone-out)	Calibration				Validation			
	
	N	Mean (kg)	Min (kg)	Max (kg)	N	Mean (kg)	Min (kg)	Max (kg)
Shoulder blade	159	4.58	3.26	6.26	154	4.40	3.37	5.49
Shoulder picnic	157	8.28	6.28	10.54	153	7.76	5.97	9.38
Loin	157	5.88	4.02	7.84	152	5.45	3.86	8.52
Belly	159	12.62	8.10	17.90	154	12.24	9.77	16.27
Ham	161	17.40	12.60	22.68	150	16.58	13.17	21.32

**Table 5:** Calibration model statistics are presented including cross-validated prediction accuracy (R2cv) and prediction errors (RMSECV)1)

Cuts (bone-out)	Calibration			Validation			
	
	N	R^2^cv	RMSECV (kg)	N	R^2^pred	RMSEP (kg)	Bias (kg)
Shoulder blade	159	0.774	0.282	154	0.486	0.268	0
Shoulder picnic	157	0.818	0.400	153	0.590	0.454	−0.25
Loin	157	0.849	0.330	152	0.744	0.413	−0.14
Belly	159	0.856	0.704	154	0.572	0.688	0.06
Ham	161	0.840	0.802	150	0.632	0.889	−0.19

R^2^cv, cross-validated prediction accuracy; RMSECV, root mean squares error of cross validation; R^2^pred, prediction accuracy of validation data; RMSEP, root mean squares error of prediction; Bias, reference values - predicted AFIII values.

1) Result of the validation test including bias (reference values - predicted AFIII values), prediction accuracy (R^2^pred) and prediction error (RMSEP) are listed for comparison.
